# Cardiopulmonary interactions—which monitoring tools to use?

**DOI:** 10.3389/fphys.2023.1234915

**Published:** 2023-08-09

**Authors:** David Berger, Per Werner Moller, Kaspar F. Bachmann

**Affiliations:** ^1^ Department of Intensive Care Medicine, Inselspital, Bern University Hospital, University of Bern, Bern, Switzerland; ^2^ Department of Anaesthesia, SV Hospital Group, Institute of Clinical Sciences at the Sahlgrenska Academy, University of Gothenburg, Gothenburg, Sweden; ^3^ Department of Anaesthesiology and Intensive Care, University of Tartu, Tartu, Estonia

**Keywords:** heart-lung interactions, volume responsiveness, monitoring, right ventricular failure, ECMO - extracorporeal membrane oxygenation

## Abstract

Heart-lung interactions occur due to the mechanical influence of intrathoracic pressure and lung volume changes on cardiac and circulatory function. These interactions manifest as respiratory fluctuations in venous, pulmonary, and arterial pressures, potentially affecting stroke volume. In the context of functional hemodynamic monitoring, pulse or stroke volume variation (pulse pressure variation or stroke volume variability) are commonly employed to assess volume or preload responsiveness. However, correct interpretation of these parameters requires a comprehensive understanding of the physiological factors that determine pulse pressure and stroke volume. These factors include pleural pressure, venous return, pulmonary vessel function, lung mechanics, gas exchange, and specific cardiac factors. A comprehensive knowledge of heart-lung physiology is vital to avoid clinical misjudgments, particularly in cases of right ventricular (RV) failure or diastolic dysfunction. Therefore, when selecting monitoring devices or technologies, these factors must be considered. Invasive arterial pressure measurements of variations in breath-to-breath pressure swings are commonly used to monitor heart-lung interactions. Echocardiography or pulmonary artery catheters are valuable tools for differentiating preload responsiveness from right ventricular failure, while changes in diastolic function should be assessed alongside alterations in airway or pleural pressure, which can be approximated by esophageal pressure. In complex clinical scenarios like ARDS, combined forms of shock or right heart failure, additional information on gas exchange and pulmonary mechanics aids in the interpretation of heart-lung interactions. This review aims to describe monitoring techniques that provide clinicians with an integrative understanding of a patient’s condition, enabling accurate assessment and patient care.

## Introduction and clinical assessment

Heart-lung interactions describe cardio-circulatory phenomena that are caused by the breathing pattern. The source of these phenomena is the coupling of the respiratory rate and heartrate, and thereby arterial and venous pulse pressures (Fisher et al., 2022). In contrast to some monitoring techniques in the field of intensive care medicine, which may be highly elaborate and technical, clinical assessment of respiratory rate and pulse pressure is simple. Several forms of heart-lung interactions are easily recognized from observation and bedside examination. An excellent clinical example is pulsus paradoxus - a drop of arterial pressure upon inspiration of more than 10 mmHg - which may occur in cardiac tamponade, severe airflow obstruction in acute asthma, tension pneumothorax, or severe RV failure. Another phenomenon is respiratory sinus arrhythmia (Sarkar et al., 2018). Palpation of the arterial pulse pressure can be aided by observing the pulse oximeter, where PPV can be visualized directly on the monitor without the need for an invasive pressure monitoring (Hartert et al., 1999). Other examples of heart-lung interactions are the reaction of the jugular vein and the arterial pulse to the Müller or Valsalva maneuvers (forced inspiration or expiration against a closed airway, respectively). In patients with decompensated left heart failure, the jugular vein collapses and the arterial pressure drops during a Müller maneuver, while a Valsalva maneuver will distend the jugular veins and increase the arterial pulse pressure (Buda et al., 1979; Marantz et al., 1990). These phenomena result from changes in intrathoracic and pericardial pressures and are physiologically linked through complex interactions with pre- and afterload (Grubler et al., 2017).

The clinician should remember that heart-lung interactions are part of normal physiology, occurring also in health. However, these phenomena are exaggerated under pathological conditions and can be provoked by changes in intrathoracic pressures, which makes them potentially useful in a clinical scenario of impaired hemodynamics. In the intensive care patient, intrathoracic pressure changes are mainly caused by mechanical ventilation. Within the framework of functional hemodynamic monitoring (Pinsky, 2014a) and depending on the clinical context, heart-lung interactions may provide diagnostic clues on the patient’s cardio-circulatory status. Advanced monitoring techniques gather information on the underlying pathophysiology of the observed phenomenon and enable the clinician to diagnose and treat the patient correctly.

## The explanatory model: optimal monitoring within the framework of venous return and cardiac function

To understand heart-lung interactions, one must briefly review Guyton’s framework of venous return (Guyton et al., 1957): the outflow from the heart is completely dependent on the inflow (Starling, 1918). The inflow—that is venous return—depends on the elastic recoil and the volume in the vascular system (Moller et al., 2019). The volume and elastic recoil create the mean systemic filling pressure (MSFP), which pushes the blood towards the right atrium (Magder, 2016). The right atrial pressure (RAP) acts as a back pressure to venous return (Moller et al., 2017). As with any system of related pressure and flow, the flow to the right atrium is opposed by the resistance to venous return (RVR) (Berger et al., 2016a; Bloch et al., 2016; Berger et al., 2019; Moller et al., 2019). The mean systemic filling pressure is the equilibrated vascular pressure of the systemic circulation at zero flow (Magder and De Varennes, 1998). A central function of the RV is to actively lower right atrial pressure to facilitate the return of blood, while the left ventricle pumps the blood back to the volume reservoir in the vascular system. This can be expressed in a simple term (VR: venous return, CO: cardiac output, MSFP: mean systemic filling pressure, RVR: resistance to venous return):

$$CO=VR=\frac{MSFP-RAP}{RVR}$$

**FIGURE 1:**
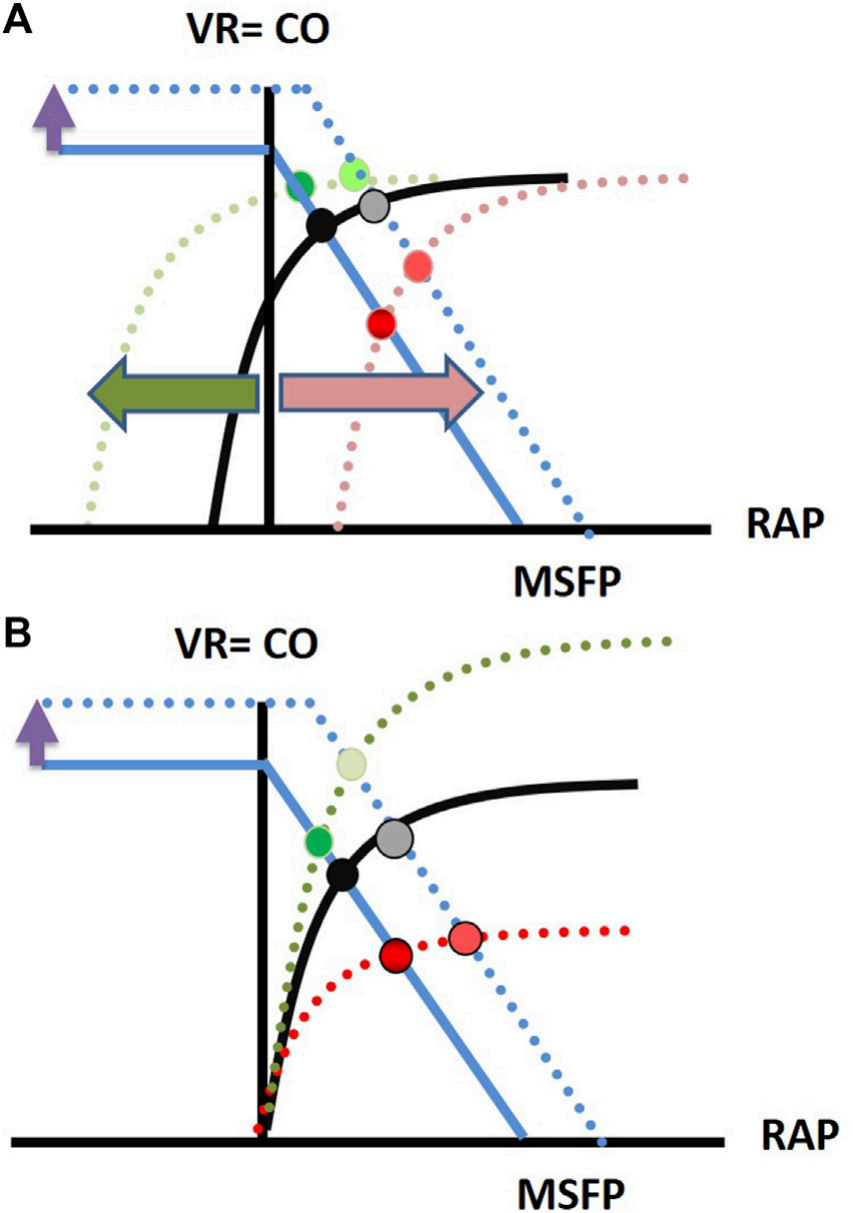
The superimposition of a Starling curve and a venous return curve provides a graphical solution to the question if a patient’s circulation is limited by venous return or cardiac function. This explains the key factors of heart-lung interactions: **(A)** Effect of volume expansion on normal Starling curve (volume responsive or limited by venous return situation) **(B)** Effect of volume expansion on flattened and steepened Starling curve (cardiac limited situation). Details are found in the text.

In a venous return curve, right atrial pressure is plotted against venous return. When venous return and cardiac function curves (Starling curve, Figure 1) are superimposed, it becomes apparent that the right atrial (or central venous pressure) serves as the equilibrium point for any given cardiovascular state. With this graphical framework, changes in inflow to the heart explain the heart-lung interactions. When right atrial pressure rises during positive pressure ventilation, venous return and thereby stroke volume must fall. The cardiac function curve shifts to the right (red arrow Figure 1A) resulting in higher RAP and lower cardiac output (and thus venous return). As the venous return curve intersects the cardiac function curve at its steep part, volume expansion (increase in MSFP, dotted blue line Figure 1A) will increase stroke volume (Moller et al., 2019). This situation would be considered as “*volume responsive*” or “*limited by venous return*”. If the venous return curve intersects the Starling curve at its flat part (higher venous return and lower RAP, green line Figure 1A), volume expansion will cause minimal increase in stroke volume. As most ICU patients are volume unresponsive (Cecconi et al., 2015), one must always consider the factors shown in panel B of Figure 1. Breath-to-breath increases in afterload (or decreases in inotropy, red dotted line Figure 1B) may flatten the cardiac response curve, particularly in scenarios of RV failure or pulmonary hypertension, and may mimic cyclic changes in stroke volume (Vieillard-Baron et al., 1999; Daudel et al., 2010; Valenti et al., 2021). Such patients would be considered “*cardiac limited*”, and their hemodynamic state would not benefit from volume expansion (no increase in venous return through rightward shift of venous return curve, Figure 1B). Administration of inotropic therapy to restore the cardiac function curve may allow the patient to benefit from increases in MSFP (green dotted line, Figure 1B). In conclusion, the extent of pulse pressure or stroke volume variation in itself is not sufficient to correctly diagnose the underlying condition and apply the correct treatment strategy (Sondergaard, 2013).

The optimal monitoring tool for the comprehensive assessment and interpretation of a patient showing heart-lung interactions would provide a prospective, combined image of venous return and cardiac function. This is not available. Of the variables necessary to plot a venous return function, only cardiac output (which equals venous return) and central venous pressure (e.g., right atrial pressure) are often assessed in shocked ICU patients. In order to fully assess the venous return function, resistance to venous return and mean systemic filling pressure should be available. Mean systemic filling pressure can be measured during circulatory standstill (Repesse et al., 2015; Repesse et al., 2017), but only surrogate measures can make their way to the bedside. Versprille and others developed a method of constructing venous return curves by progressively increasing airway pressures with consecutive inspiratory hold maneuvers (Versprille and Jansen, 1985; Hartog et al., 1996), which allows to extrapolate mean systemic filling pressure. The method was later refined by the research group of Maas and others (Maas et al., 2009; Maas et al., 2011; Maas et al., 2012; Persichini et al., 2012; Wijnberge et al., 2022). With this method, the expected hemodynamic responses according to the physiological framework of venous return, could be confirmed following volume expansion and vasoconstriction in patients after cardiac surgery, and during septic shock (Maas et al., 2009; Wijnberge et al., 2022; van den Berg et al., 2002; Aya et al., 2017; Cecconi et al., 2013), but the extrapolated values of MSFP may be inaccurate (Berger et al., 2016b). The underlying volume state (i.e., the state of interest) influences the accuracy of the measurements (Berger et al., 2016b; Werner-Moller et al., 2019), which may render this method invalid at the bedside (Moller and Berger, 2023). Parkin and Leaning developed a simple formula to calculate an analogue for mean systemic pressure (Pmsa) based on the readily available variables of right atrial pressure, mean arterial pressure and cardiac output (Parkin and Wright, 1991; Parkin et al., 1994; Parkin, 1999; Parkin and Leaning, 2008; Sondergaard et al., 2016). The method also allows for quantitative assessment of heart efficiency and the effect of volume expansion (Gupta et al., 2015; Sondergaard et al., 2016) and was validated with good accuracy and precision against a zero-flow reference method (Werner-Moller et al., 2022).

## Monitoring of arterial and central venous pressure swings

One of the first descriptions of heart lung interactions goes back to the 18th century, when Sir Stephen Hales placed a glass tube into the jugular vein of a mare and observed a cyclic change of the blood column during respiration (Sette et al., 2012). Ever since, invasive pressure measurements have been a cornerstone for heart-lung interactions, where the pulse pressure variation serves as a surrogate for the stroke volume variation caused by heart-lung interactions. As invasive monitoring of central venous and arterial pressure still constitutes the best practice standard for shocked patients in the ICU (Cecconi et al., 2014), these swings will be readily observable, with most research interest and clinical attention given to the arterial pressure. However, interpretation is only possible in patients without spontaneous respiration (Jardin, 2004; Magder, 2004). Most commonly, the arterial pressure swings are taken as signs of overt or relative hypovolemia or volume responsiveness. At this point, we must remember *a first caveat*: a circulation which is not cardiac limited will always be fluid responsive (Magder, 2004). Fluid responsiveness is neither a pathological condition, nor does it indicate that fluid administration is necessary or beneficial. The question of fluid responsiveness must be preceded by a clinical assessment of whether fluid expansion and/or increased flow is at all warranted (Magder, 2004; Takala, 2016).

Based on experimental work by Morgan et al. (1966), Perel et al. (1987), Michard et al. (2000) showed in a landmark paper that arterial pulse pressure variability over the respiratory cycle predicted fluid responsiveness (defined as a pulse pressure variability of >13% for an increase in cardiac output of 15%) in septic patients with circulatory failure.

Pulse pressure variation (PPV) is a dynamic test: a reproducible change in the pleural pressure over the respiratory cycle elicits a response from the cardiovascular system. The introduction of dynamic testing was a major advance, since static one-point measurements like central venous or pulmonary artery occlusion pressure was proven unreliable for the prediction of volume responsiveness (Osman et al., 2007; Marik and Cavallazzi, 2013). Assessment of PPV inspired a high research interest and was developed further to include additional monitoring modalities like stroke volume variability (SVV) from pulse contour analysis or analysis of pulse-oximetry plethysmograms, and specific maneuvers to increase the diagnostic yield, discussed below.

The central physiological rationale behind these techniques is the cyclic increase in RAP caused by an increase in pleural pressure from mechanical inspiration. This immediately lowers venous return (Moller et al., 2017) and RV stroke volume. This smaller stroke volume is forwarded to the left ventricle, where the lower pulse pressure appears during expiration. This idea fits well with the isolated concept of venous return, but it neglects all factors from the right heart, cardiac valves and pulmonary factors (Sondergaard, 2013). The meta-analytic pooled sensitivity and specificity reached almost 90% for volume responsiveness (albeit with tidal volumes larger than 8 mL/kg, which is not currently standard of care) (Yang and Du, 2014) and may be increased with provocation maneuvers. In situations of lung protective ventilation, a stepwise increase of the tidal volume (tidal volume challenge) or the end-expiratory occlusion test may increase the diagnostic performance for pulse pressure or stroke volume variation (Monnet et al., 2009; Wang et al., 2023).

The critical care physician is well advised to interpret PPV and provocation tests, with *a second caveat*: as the entire pulmonary compartment including the RV lies before the measurement of systemic arterial PPV, it signals a preload dependency of the left ventricle (Magder, 2007) - but not of the entire circulation. Factors in the lung like atelectasis, changes in tidal volumes, hypoxic vasoconstriction, and pulmonary hypertension may blunt PPV while RV failure may cause PPV, but further volume administration in RV failure can be detrimental (Sondergaard, 2013; Slobod et al., 2022). Therefore, RV failure and pulmonary hypertension render PPV invalid for the prediction of volume responsiveness (Daudel et al., 2010; Wyler von Ballmoos et al., 2010). In such context, PPV and related techniques may rather reflect an afterload-dependency of the RV (Vieillard-Baron et al., 1999; Vieillard-Baron et al., 2015; Valenti et al., 2021). Several pulmonary factors like compliance (Robotham et al., 1983; de Waal et al., 2009; Mesquida et al., 2011), respiratory rate, tidal volume (De Backer et al., 2005; De Backer et al., 2009; Slobod et al., 2022), and airway driving pressure (Muller et al., 2010) influence PPV, as will circulatory factors like the need for a sinus rhythm and aortic elastance and thus ventricular-aortic coupling (Magder, 2010; Sondergaard, 2013). Since the introduction of PPV derived methods at the bedside, a complex series of clinical and physiological conditions were identified as relevant influences. The clinician should account for these factors before taking decisions based on PPV (Sondergaard, 2013; Vieillard-Baron et al., 2016). In order to assess PPV within an integrative picture, additional information (e.g., specific monitoring) is needed.

Besides invasive arterial pressure, invasive measurement of central venous or right atrial pressure are routinely available (Cecconi et al., 2014). A single static measurement of CVP neither predicts blood volume nor volume responsiveness (Marik and Cavallazzi, 2013), but the CVP is the equilibrium or interaction point of the cardiac and venous return function. When understood as such, changes in CVP over time or during specific maneuvers may provide valuable information: a healthy RV will keep central venous pressure low. An increasing CVP therefore reflects a declining cardiac function, and/or an increased venous return from increased blood volume or vasoconstriction (Magder, 2017). A rapid, sustained rise in CVP from a volume bolus, without increase in stroke volume, is a strong indicator of right heart dysfunction and an important safety limit against further volume expansion (Magder, 2017). The inspiratory drop of CVP is one of the few measurements that allow estimation of fluid responsiveness in the spontaneously breathing patient. The absence of an inspiratory negative swing makes fluid responsiveness unlikely (high negative predictive value), but the positive predictive value is poor (Magder et al., 1992; Magder, 2017).

We have now an integrated assessment of arterial and central venous pressure - one measured downstream and the other upstream of the heart. If these measurements cannot identify the relevant factors to assess the clinical situation, the use of more advanced monitoring techniques is necessary.

## Advanced bedside techniques: pulmonary artery catheterization, transpulmonary thermodilution, and echocardiography

Even though use of the pulmonary artery catheter (PAC) has declined over the last decades after a series of negative clinical trials (Parker et al., 2023), it remains a cornerstone for complex hemodynamic monitoring and is still recommended for situations with impending right heart failure and for patients unresponsive to initial treatment (Vieillard-Baron et al., 2016). As increases in RV afterload are common in pulmonary diseases and may exacerbate heart-lung interactions (Vieillard-Baron et al., 1999; Schmitt et al., 2001; Vieillard-Baron and Jardin, 2003; Repessé et al., 2016; Valenti et al., 2021), the PAC’s ability to monitor RV dysfunction with an increased ratio of CVP to pulmonary artery occlusion pressure is helpful (Monchi et al., 1998; Osman et al., 2009). Additionally, since disturbed gas exchange may contribute to hypoxic pulmonary vasoconstriction - often presenting as an increased transpulmonary pressure gradient (Bull et al., 2010) and an increased isovolumetric contraction pressure in pulmonary artery pressure tracings (Jardin et al., 1987; Jardin et al., 1989; Berger et al., 2014; Slobod et al., 2022) - the continuous assessment of mixed venous oxygenation can help assess the extent of vasoconstriction and shunt, and the contribution of venous admixture to oxygenation disorders (Takala, 2007; Bootsma et al., 2022). The pulmonary artery pulsatility index has rarely been studied for heart-lung interactions but offers a theoretically promising tool (Lim and Gustafsson, 2020; Bootsma et al., 2022). A shortcoming of the PAC—stemming from the thermodilution technique - is its inability to report immediate changes in stroke volume. However, pulmonary pulse contour analysis with direct assessment of RV stroke volume is feasible (Berger et al., 2020) and this limitation may be overcome by advanced PAC models with shorter thermodilution response times (Bootsma et al., 2022).

Monitoring tools that enhance the accuracy of continuous pulse contour analysis by intermittent calibration using transpulmonary thermodilution offer the advantage of beat-to-beat stroke volume assessment. They also report volumetric preload parameters like global end-diastolic or intrathoracic blood volumes (Reuter et al., 2002; Reuter et al., 2010). Importantly, both derive from a (common) global measure of indicator distribution volume and as such are unable to distinguish between anatomical structures of the right and left heart. Therefore, the technique cannot in itself recognize right heart failure, which is the main imitator of fluid responsiveness.

Critical care echocardiography has become an indispensable bedside tool for hemodynamic assessment, both for diagnosis and monitoring (Vieillard-Baron et al., 2019). Doppler echocardiography with measurements of pulmonary venous flow velocity and left ventricular (LV) velocity time integrals contributed significantly to the elucidation of the pathophysiology of heart-lung interactions. The inspiratory increases in arterial pulse pressure are caused by increased LV filling (Vieillard-Baron et al., 2003). For the RV, an inspiratory drop of stroke volume is caused by increased afterload (Vieillard-Baron et al., 1999).

Various dynamic echocardiographic tests were developed to predict fluid responsiveness: change in the diameter of the superior vena cava over the respiratory cycle has been proposed as a volume gauge (Vieillard-Baron et al., 2004), but it is influenced by thoracic compliance and may become decoupled from stroke volume in cases of high RV afterload (Lansdorp et al., 2014; Valenti et al., 2021). Diameter changes in the inferior cava are not valid in situations of increased abdominal pressure (Vieillard-Baron et al., 2018a). The sensitivity to predict fluid responsiveness for both methods is moderate: Vignon et al. (2008) demonstrated—in a direct comparison of both caval vein methods—“classic” arterial pulse pressure variation and changes in the aortic flow velocity, that respiratory changes of the maximum aortic flow velocity had the highest diagnostic yield and outperformed invasive pulse pressure variation for the prediction of fluid responsiveness (Vignon et al., 2017). VEXUS (venous excess ultrasound score) is a relatively new approach, not based on dynamic indices, that may allow assessment of a patient’s volume state in relation to the right ventricular function. It is based on IVC diameter and the flow pattern of hepatic, portal, and renal veins as well as renal arterial resistive index and grades the level of venous congestion (Argaiz et al., 2022; Longino et al., 2023). This promising tool may add to the integral assessment of a patient’s hemodynamic condition but needs further validation in critically ill patients.

Echocardiography is also a key modality for the diagnosis of diastolic dysfunction. The role of diastolic dysfunction for heart-lung interactions is unclear. Since all echocardiographic parameters for diastolic dysfunction depend on cardiac loading conditions, this interdependency should be evaluated in future research projects (Vignon et al., 2007; Juhl-Olsen et al., 2012; Juhl-Olsen et al., 2013; Berger et al., 2022a; Berger et al., 2022b).

A particular strength of echocardiography is its ability not only to *monitor* hemodynamics, but to diagnose specific conditions like tamponade or acute core pulmonale, which may aggravate or mimic heart-lung interactions (Vieillard-Baron et al., 2018b; Vieillard-Baron et al., 2020). The major differential diagnosis for the occurrence of heart-lung interactions is volume responsiveness vs. right heart failure (Vieillard-Baron et al., 2016), or a preload-vs. an afterload problem (18). This includes the visualization of the diastolic interventricular dependency with leftward septal shift and increased right ventricular and decreased left ventricular end-diastolic volumes as key components for the understanding of why right ventricular failure may lead to PPV. Here, echocardiography plays a crucial role, but the clinician must remember *several caveats*: there is no common echocardiographic definition of RV failure (Vieillard-Baron et al., 2018c). Standard measures like TAPSE may not be applicable in critically ill patients (Vieillard-Baron et al., 2020). Since the RV works below its stressed volume (Pinsky et al., 1992; Pinsky, 2014b; Bachmann et al., 2020), RV dilation is a normal adaption to increased load, until the compliance reserve is exhausted and central venous pressure rises. Table 1 summarizes the diagnostic approaches.

**TABLE 1:** Monitoring tools presented within their respective category and their advantages and disadvantages. Distinguishment between preload and afterload causes of PPV as well as degree of invasiveness were rated by the authors using the following scale (--) strongly disagree, (−) partially disagree, (0) no statement possible, (+) partially agree, (++) strongly agree.

Monitoring tool	Parameters	Advantages	Disadvantages	Distinguishes preload and afterload causes of PPV	Degree of invasiveness
**Pulse palpation**	Qualitative assessment of pulse pressure	Readily available	Observer dependent, no quantification of PPV, does not allow differentiated interpretation	--	--
**Jugalar vein distension/collapse**	Qualitative assessment of preload conditions	Readily available	Observer dependent, no quantification of PPV, does not allow differentiated interpretation	--	--
**Oxymetry swing**	Qualitative assessment of pulse pressure	Readily available	Observer dependent, no quantification of PPV, does not allow differentiated interpretation	--	--
**Right atrial pressure/central venous pressure**	Quantitative assessment of right ventricular diastolic function and backpressure to venous return	Readily available in the ICU. Reflects the equilibrium point of the cardiac function curve and venous return curve	Static measurement does not allow assessment of fluid responsiveness. Measurement may be inaccurate if not leveled precisely	-	+
**Mean systemic pressure analogue, Pmsa**	Quantitative assessment of the volume state (i.e., stressed volume), venous return driving pressure, and derived variables such as heart efficiency [Eh=(Pmsa-CVP)/Pmsa]	Allows assessment of the systemic volume state, the venous return function, and a derived global measure of heart efficiency	Calculated value dependent on multiple, possibly inaccurate measurements (CVP, ABP, CO)	+	++ (CO necessary)
**Pulse pressure variation (systemic)**	Quantitive assessment of pressure swings in arterial tracing	Readily available in the ICU. Values are given in percentages and can be traced over time	Assessment between right ventricular failure and low volume states may be difficult	--	+
**Pulmonary artery catheter**	Quantitative assessment of afterload conditions (PAP), left ventricular diastolic function (LVEDP), cardiac output and right ventricular ejection fractions	Full assessment of right ventricular function and afterload conditions as well as left ventricular filling pressures; may help detect causes of cardiogenic shock if applied correctly	Not readily available and invasive procedure with associated risks if not applied regularly	++	++
**Transpulmonary thermodilution**	Quantitative assessment of cardiac output, pulse contour analysis (stroke volume) and volumetric measure of central filling conditions	Accuracy of stroke volume assessment by pulse pressure contour analysis is enhanced by intermittent calibration using thermodilution. A volumetric measure of central volume state is appealing	Indices are global and do not allow differentiation of causes of PPV. May become inaccurate at extreme conditions	-	+
**Echocardiography**	Qualitative and quantitative assessment of biventricular systolic and diastolic function, structural abnormalities including valvular function and cardiac output	Comprehensive assessment of biventricular systolic and diastolic function, diagnosis of relevant structural abnormalities. Allows quantitative assessment of loading conditions and output	Point-of-care assessment without option to monitor cardiovascular function continuously. Does not give information on vascular state and venous return function. Indices may be load dependent	+	--
**Vexus Score (Ultrasound)**	Qualitative assessment of volume state with regards to right ventricular dysfunction	Allows assessment of right ventricular filling conditions in combination with volume state. May facilitate deresuscitation and can be tracked over time	No continuous assessment. Lacks validation in large ICU cohorts	++	--

## Conclusion

Heart-lung interactions provide valuable insights into the pathophysiology of patients, with the potential to provide crucial information for personalizing treatment. The best choice of monitoring tool depends on several factors, including the clinical scenario, the local accessibility and proficiency of a specific technique, as well as the familiarity, training, and confidence of the clinician in utilizing a particular method. Optimal information can be obtained through the integration of multiple sources, employing dynamic testing and trend analysis, and gradually advancing the monitoring process if the initial treatment proves ineffective. When uncertainty arises, it is essential to actively consider the possibility of RV failure.
